# Committees Appointed to Report at the Next Meeting of the American National Association

**Published:** 1847-07

**Authors:** 


*Committees Appointed to Report at the next Meeting of the American National Association.*—The following are the standing Committees, appointed by the President and Vice-Presidents of the Medical Association, to report at its next meeting, at Baltimore, on the first Tuesday in May, 1848, as ordered by the Constitution of the Association :—

*Committee on Arrangements.*

Dr. G. C. M. Roberts, Balt., Chairman.

Dr. A. C. Robinson, Balt.

" J. H Briscoe, "

" J. R. W. Dunbar, "

Dr. Wm. Power, Balt.

" W. T. Leonard, "

" C. Bell Gibson, "

*Committee on Medical Sciences.*

Dr. S. Henry Dickson, S. C. Chairman.

Dr. J. P. Jervy, S. C.

" Robert Bridges, Phil.

" J. W. Francis, N. Y.

Dr. Wm. T. Wragg, S. C.

" Wm. Power. Balt.

" T. Romeyn Beck, N. Y.

*Committee on Practical Medicine.*

Dr. Joseph M. Smith, N. Y. Chairman.

Dr. Rene La Roche, Phil.

" John Harrison, La.

" H. M. Bullitt. Mo.

Dr. J. Beck, N. Y.

" Isaac Wood, "

" G. S. Camman, "

*Committee on Surgery.*

Dr. George W. Norris, Phil., Chairman.

Dr. Isaac Parrish, Phil.

" John Watson, N. Y.

" A. L. Pierson, Salem, Mass.

Dr. Jacob Randolph, Phil

" H.H. M'Guire, Petersburg, Va.

" C. Bell Gibson, Balt.

*Committee on Obstetrics.*

Dr. Harvey Lindsley, D. C. Chairman.

Dr. G. C. M. Roberts, Balt.

" J. Riley, D. C.

" R. W. Haxall, Richmond, Va.

Dr. W. Channing, Boston.

" C. R. Gilman, N. Y.

" S. Annan, Lexington, Ky.

*Committee on Medical Literature.*

Dr. Oliver Wendell Holmes, Chairman.

| | |
|---|---|
| Dr. E. Hale, Boston. | Dr. John Bell, Phil. |
| " G. C. Shattuck, Jr., " | " Austin Flint, Buffalo. |
| " D. Drake, Louisville, Ky. | " W. Selden, Norfolk, Va. |

*Committee on Medical Education.*

Dr. Alexander H. Stevens, Chairman.

| | |
|---|---|
| Dr. Amos Twitchell, Keene, N.H. | Dr. R. D. Arnold, Savannah. |
| " B. R. Wellford, Fredericksburg, Va. | " F. Campbell Stewart, N. Y. |
| " Arnold Naudain, Phil. | " L. P. Bush, Wilmington, Del. |

*Committee on Publication.*

Dr. Isaac Hays, Phil., Chairman.

| | |
|---|---|
| Dr. Alfred Stille, Phil. | Dr. J. R. W. Dunbar, Balt. |
| " J. V. C. Smith, Boston. | " Gouverneur Emerson, Phil. |
| " J. P. Garvin, Augusta, Ga. | " Caspar Morris, " |

*Committee on Indigenous Botany, under the Resolution of Dr. N. S. Davis.*

Dr. N. S. Davis, Binghamton, N. Y. Chairman.

| | |
|---|---|
| Dr. S. W. Williams, Mass. | Dr. A. C. Robinson, Md. |
| " Eli Ives, Conn. | " Frederick Marx, Va. |
| " Engleman, Mo. | " J. P. Porcher, S. C. |
| " W. A. Cheetham, Tenn. | " J. Le Conte, Ga. |
| " Jos. Carson, Pa. | " Cartwright, Miss. |
| " Charles Short, Ky. | " Carpenter, La. |
| " E. E. Phelps, Vt. | " John M. Bigelow Ohio. |
| " A. Twitchell, N. H. | " G. Norwood, Ind. |
| " T. C. Dunn, R. I. | " Merryman, Ill. |
| " Lyndon H. Smith, N. J. | " Russell, Mich. |
| " James Couper, Del. | " J. Riley, D. C. |

